# On Wounds from Fire-Arms without Ball

**Published:** 1847-04

**Authors:** 


*On Wounds from Fire-Arms without Ball.* By Paul Swift, M. D., Philadelphia.

There is obviously a wide spread popular error in relation to the effect of the explosion of gunpowder without ball ; and even professional writers on this subject are not very definite in regard to the distance at which a pistol, or other fire-arm, so loaded, may be discharged, without inflicting a dangerous wound.

The popular notion and language is, that the piece is not loaded unless it be charged with ball, slug, or shot, as well as powder, and that its discharge is quite safe even when held near the person.—Moreover, when wounds do occur from such discharges, all parties seem quite sure that the *wad* is the immediate cause. The following case will illustrate the prevalence of this opinion and its fallacy:

On last New-Year's eve, at the Good Will Engine house, Wm. Simler, a minor, playfully, but heedlessly, fired a pistol charged with powder only, at his friend and companion Robert W. Pitt, inflicting a serious wound. Pitt staggering into the arms of his friends, cried out, "I am shot." Simler, thinking him frightened, but not harmed, said, laughing, "It was not loaded," or, as another witness testified, "It had no ball in it." The wound was on the fleshy part of the left hip, above and behind the trochanter major, about one inch in diameter, and four inches in depth; the integuments were destroyed, and the muscles presented a blackened, mangled mass: it bled but little, and was carefully probed with the finger, which readily passed to the bottom of the wound. No untoward symptom arose till the sixth day, when tetanus in its most distressing form, opisthotonos, supervened, and he died on the morning of the seventh.

On a careful post-mortem examination, no foreign substance was found but a minute fragment of woolen cloth, about two inches from the surface, and *grains of gunpowder, with which the wound, through its whole extent, was blackened.*

Thus it was evident that this fatal wound was caused by the explosion of gunpowder in a pistol of ordinary size; a wound at least four times as large as a ball from the same instrument would have caused; and so mangled were the tissues, through this great extent, that vitality was utterly destroyed. Had the unfortunate young man lived, it must have been through great suffering, necessarily attending the tedious and exhausting sloughing of the dead mass.

At the legal investigation which followed, there was some discrepancy in the testimony in regard to the distance at which the pistol was held when the wound was inflicted, the witnesses differing from one foot to two or three yards; nor is this very strange, as it occurred in the night, in a place not well lighted, and in the midst of a moving throng of some twenty individuals. The patient himself, however, declared his belief that the weapon "almost touched him." The pistol was said, by Simler, to have had a paper wad; but no wad was at any time found, and the evidence given at the time rendered it quite probable none was used.

Being one of the professional attendants in this case, I was somewhat surprised at the character and extent of the wound; it was obvious no ball could have produced it; nor was it conceivable that a paper wad could have caused such extensive lesions. A considerable research in works on medical jurisprudence failed to give satisfaction, and though the circumstances forced upon my mind the inference that a heavy charge of powder, exploded near the part, had alone caused the mischief, yet, as young Simler had been bound over for trial before a criminal court, it seemed very desirable to possess farther data in the premises. With this view I sought, and through the courtesy of the officers of Jefferson Medical College, obtained the facilities for making the following experiments, which were used at the subsequent trial, and are now offered to the public with a hope that they may be useful in future medico-legal investigations of this nature. The pistol—the same used by Simler—was wadded with paper, and has a bore about four inches in depth, and six lines caliber.

*Experiment 1st.*—Pistol with an ordinary charge was held 12 inches from fleshy part of hip, the part being covered with one thickness of broadcloth and a twilled cotton cloth under it—Clothes lacerated, and skin abraided ; wad on the floor, on fire.

*Experiment 2d.*—Distance 6 inches ; parts covered as before, clothes lacerated ; wad lodged one inch and a half below the surface.

*Experiment 3d.*—Distance 2 inches—wound ragged, blackened with powder, and penetrating to the bone—one and a half to two inches—wad was found immediately beneath the integuments, and somewhat *on one side of the principal wound*—parts covered as before.

*Experiment 4th.*—Distance one and a half inch from the *ribs* of the right side—no covering of cloth—penetrated the cavity of the chest, the wad passing through the intercostals between the ribs.

*Experiment 5th.*—Distance the same—no covering of cloth, the integuments removed—wad penetrated the thorax, *carrying away a transverse portion of the rib.*

The subject, about 35 years of age, a male, not recent, had undergone a preserving process with chloride of mercury, considerably hardening the muscles ; it was also much emaciated.—*Medical Examiner.*

