# Erratum in the Article on Wounds from Fire Arms without Ball, Contained in the Eclectic Department of This No

**Published:** 1847-04

**Authors:** 


*Erratum in the article on wounds from fire arms without ball, contained in the Eclectic Department of this No.*—We find in a No. of the Medical Examiner received since the article above referred to was printed, a communication from Dr. Swift, correcting a material error. We accordingly publish the letter :

*To the Editor of the Medical Examiner.*

Dear Dr.—In my communication in the last No. of the Medical Examiner, on Wounds from Fire-arms without Ball, the 2d Experiment is thus reported : "Distance six inches, parts covered as before, clothes lacerated, wad lodged one inch and a half below the surface."

This is a material error ; it should read, *one half inch* below the surface; and as the experiment may possibly be referred to in future medico-legal inquiries and criminal prosecutions, thou wilt oblige me by giving this correction in the next number of the Examiner.

Respectfully and truly, thy friend,

Paul Swift.

Philadelphia, 3d mo. 11th, 1847.

